# MENTAL HEALTH RESILIENCE IN SWISS OLDER ADULT SURVIVORS OF CHILD WELFARE–RELATED MALTREATMENT

**DOI:** 10.1093/geroni/igac059.1672

**Published:** 2022-12-20

**Authors:** Myriam Thoma, Florence Bernays, Andreas Maercker, Shauna L Rohner

**Affiliations:** University of Zurich, Zurich, Zurich, Switzerland; University of Zurich, Zürich, Zurich, Switzerland; University of Zurich, Zurich, Zurich, Switzerland; University of Zurich, Zurich, Zurich, Switzerland

## Abstract

Minors affected by child welfare practices in Switzerland during the last century had a high risk for exposure to childhood trauma and maltreatment. Several studies with this cohort demonstrated substantially higher levels of clinically-relevant psychopathology in older adult survivors in comparison to non-affected control individuals. However, these studies also revealed that not all affected individuals developed mental health disorders over their lifespan. To date, this mental health resilience in survivors of an advanced age is still insufficiently understood. Therefore, this study aimed to assess and compare the resilience profiles of older adults who were formerly affected by child welfare-related trauma and maltreatment (risk group, RG; n = 132; Mage = 71 years) and non-affected, age-matched controls (control group, CG; n = 125). Within the RG, approximately one-third of the individuals had no current or lifetime DSM-5 mental health disorders. In comparison to the survivors with a history of mental ill-health, these individuals were older, had a higher income, and expressed a higher subjective satisfaction with their socio-economic status. Furthermore, they reported less early-life physical abuse, and had lower levels of neuroticism, as well as empathy-related characteristics. In addition, they showed higher levels of self-esteem and trait resilience. Group differences between the RG and CG highlight the importance of considering past adversity in the understanding of mental health resilience in later life.

